# Interview with Dr Alastair Santhouse

**DOI:** 10.1192/bjb.2025.10167

**Published:** 2026-04

**Authors:** Abdi Sanati

**Figure:**
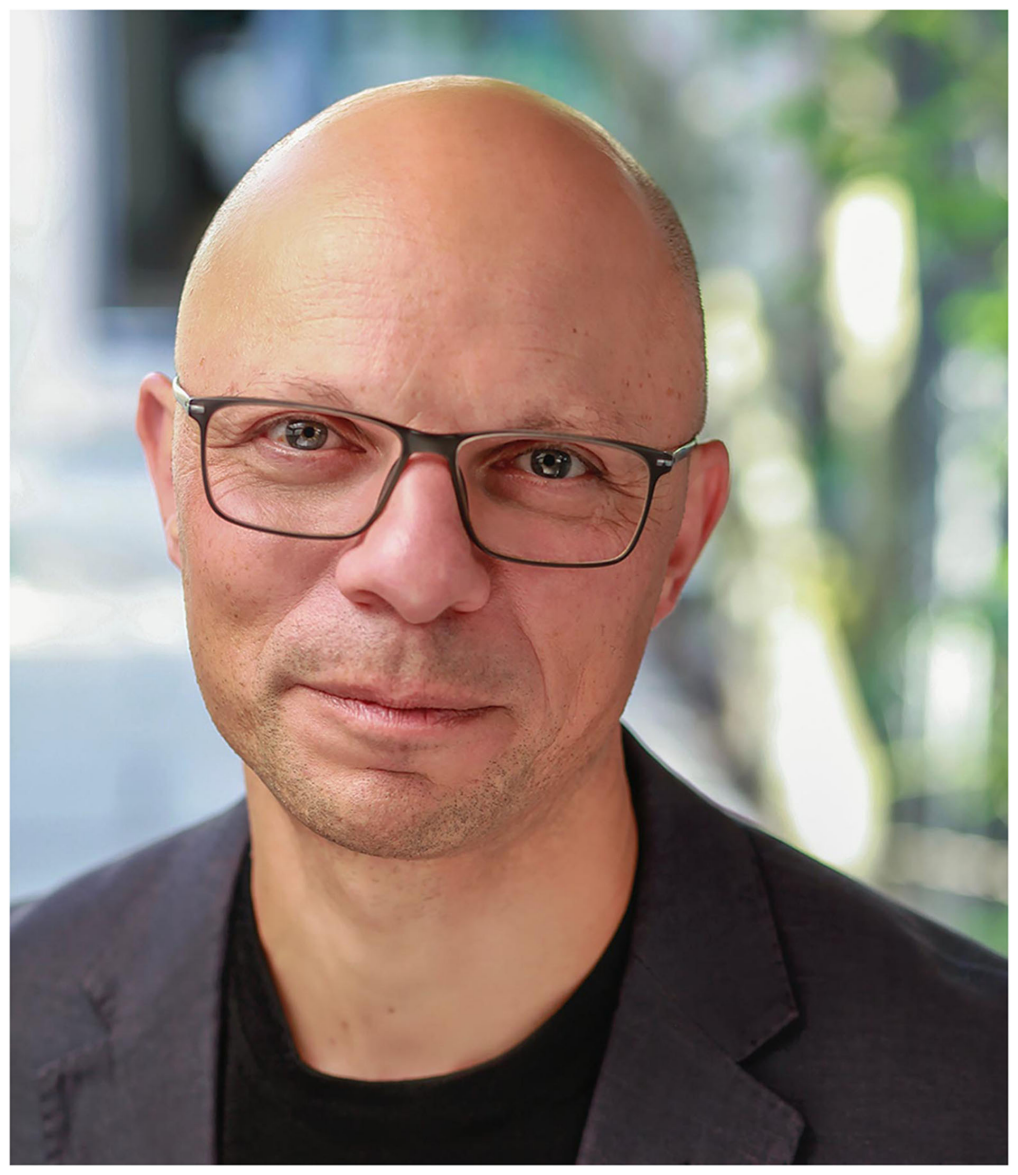

Dr Alastair Santhouse is a consultant neuropsychiatrist in London. He is a Fellow of both the Royal College of Psychiatrists and Royal College of Physicians. I first met Dr Santhouse in 2001. At that time, I was sitting the MRCPysch Part 1 exam. In the good old days it was done differently! For preparation, I attended the Manchester course which Dr Santhouse organised alongside another colleague. I found Dr Santhouse’s teaching engaging and very informative. As I progressed further our paths crossed from time to time. He is a very well read, intelligent and well informed person, in and out of psychiatry. Recently I came across his new book, *No More Normal: Mental Health in an Age of Over-Diagnosis*, which shows how he continues to be an excellent communicator, informing the reader of the challenges psychiatry faces in 21st century, without descending into anti-psychiatry. I caught up with Dr Santhouse after many years, when apart from giving me his time for the interview, he advised me on the best translation of *The Master and Margarita*, one of his favourite books!

**Thank you very much for your time for the interview. I enjoyed reading your book very much. My first question is: what motivated you to write the book?**

That’s a really good question. It came from an observation that over recent years we have seen a substantial rise in the number of mental health diagnoses. It is apparent not just in the wider statistics, but also evident in my clinics, with people wondering about or identifying with various diagnoses, often the same diagnoses. It has been, I think, an escalating trend over perhaps the last 10 to 15 years. I wanted to try and understand this change and what accounted for it. So, the motivation in writing the book was in trying to make sense of what I was seeing in my clinics every week and in society more generally.

**In the book you mention the change in terminology from ‘mental illnesses’ to ‘mental health conditions’. I remember in 2016 our former president Professor Dinesh Bhugra wrote an article reclaiming the term ‘mental illness’.** ^1^ **Do you think that this change in terminology has had some effect on the way that we practice?**

I think it has added to the confusion about what we mean by the term ‘mental illness’. If someone says that their mental health isn’t good, are they telling you that they’ve got a mental illness, or are they telling you something else by it? It is a little unclear. And I think we are in an age of a certain confusion about where the boundaries of mental illness are and what might be conceived differently as suffering or distress. These may be very difficult or even incapacitating for an individual but do not need a mental health pathway. People do need help, but it needs to come from somewhere else and not necessarily from the GP or mental health services prescribing medications.

**You mentioned that people are seeing diagnosis as a form of identity. Do you think that the way that these diagnoses have become identity labels is useful for some people?**

The most important thing is whether a diagnosis is correctly made. In other words, is the label appropriate and true? Where it is, and in the sense of people understanding or making sense of their lives through a diagnosis, I think it can be helpful.

But my concern is that many diagnoses are made without adequate consideration or are self-diagnoses, and here I believe diagnostic labels can do harm. In some people they can becoming limiting, can change self-perception and induce a feeling that they are no longer in control. They may also be encouraged to go down a psychiatric treatment pathway that isn’t appropriate for their needs. We are not supporting individuals to get the help that they need, by the people that are in the best place to deliver it.

**Could it be possible that this anti-stigma campaign, or mental health awareness campaign, has inadvertently contributed to this?**

I think everyone would agree that reduction in stigma has to be a good thing, and that a more knowledgeable, better-informed public is a positive thing too. And yet, somehow, we have got to a position where the wider mental health profession is too ready to give out diagnostic labels, sometimes without adequate expertise, perhaps paralleling people’s increased acceptance of them.

You could argue that acceptance of labels because of a reduction in stigma is progress – but on the other hand, isn’t that what anti-stigma campaigns always used to be about? That you were more than simply a diagnostic label?

**You have phrased it in terms of over-pathologising. If someone asks you what is wrong with over-pathologising, what answer would you give to them?**

Well, let’s take trauma as an example. Imagine someone comes to you with a life experience including adversity, but these adversities don’t cross that threshold that we would typically understand as being necessary for a post-traumatic stress disorder label.

If you tell them they were traumatised, whatever that means, you may be just adding to their difficulties by telling them that they’ve got a problem now that is a pathological issue that they are not any longer able to resolve on their own. I see this a lot. I think it might change someone’s ability to believe that they can manage the problem that they have or control it. On the face of it, it may seem kind, supportive or empathetic to tell someone that they have been traumatised, but in many cases I don’t think it is helping them. Giving a diagnosis is a big step. It needs to be done carefully and with mature judgment. And I feel in a lot of cases people are very quick to move to a diagnosis.

**It is interesting that you mentioned trauma. We have seen a rise of trauma. I have heard people saying that everything could be explained in terms of trauma. Why do you think that trauma has become so big?**

Many people lead difficult lives of real adversity and suffering. I certainly would not want to deny or understate that. Yet one of our contemporary issues is that there is very little consensus about which of these count as trauma. I know what the ICD classification would say, but people are not really using that. It has become something of a self-defined concept. Trauma might be a helpful sort of language for people to frame someone’s difficulties. The problem is that once you have that framing, you can get stuck in it.

**Going back to over-pathologising. What role do you think that the classification systems like DSM and ICD have in this process?**

This is a really important issue. I think that our classification systems in many areas are not doing a good enough job of differentiating what is a variation of normality from what is pathological. So, there can be a confusion about where that boundary is. This includes existing diagnoses as well as new diagnoses. As a profession I think we need to look at ourselves and ask are we doing a good enough job.

**Another thing you mentioned in your book is the kind of difference between categorical models and dimensional models. Now, thinking about it, one thing that came to my mind was that a kind of non-categorical model could be more efficient in pathologising. Now, I just couldn’t help but remember what Jung said: show me a sane man, and I will cure him for you. What is your opinion?**

The nature of diagnoses and how we make them is central to the issue we have in psychiatry. We can imagine two extremes. At one end, the argument would be that mental illnesses are real entities, existing somewhere in our brain, with the underlying pathology and brain networks waiting to be discovered.

At the opposite end of the spectrum, the theory would be that what we refer to as mental illness is just a social construct: essentially, artificial labels given to people who do not fit into societal norms.

There are of course lots of positions somewhere between these two extremes, and I suspect that different mental illnesses occupy different parts of that spectrum. That is the problem that psychiatry wrestles with, which is, in the absence of any clear-cut pathological findings or reliable investigations, how do we conceptualise mental illness? At the moment, people are defined by their own symptoms, and we need to try and make sense of that. Some are much easier than others. But for dimensional diagnoses, like depression, it can be difficult. Where is mild depression? What is normal sadness? It is not always an easy determination to make.

**One thing you mentioned in the book is the diagnosis as a product or consumer good, and people going to the psychiatrist for a diagnosis as something they buy. But, having said that, this kind of market economy has been around for a long time. Why do you think that it is only recently that this has become a product, or commodity?**

I don’t think it is necessarily quite as transactional as that, but I think the way our society is constructed is that very often you need a diagnosis to access the help that arguably should be available without someone needing to have a diagnosis. So, for example, someone struggling at school or in the workplace might need a formalised diagnosis to be able to access help they need. There are other reasons. People are understandably trying to make sense of their lives and often their suffering, too. In different eras and different generations, people had a different way of doing this. The framing of suffering as mental illness seems to be a contemporary way of conceptualising this and has led to a far greater interest and acceptance of mental health labels.

**You have also written about risk. I believe that we have got ourselves into a quagmire by thinking we can predict the future. Could you explain your opinion on how risk has dominated our work?**

Well, over the time – almost 30 years – that I’ve been a psychiatrist, one of the biggest changes that I have seen is the bureaucratisation of risk. Risk is, of course, something that we clearly need to be thinking about and managing. But we ended up trying to do it by increasingly complex forms and numerical scoring.

We are finally moving away from risk scores, but it remains the case that risk management is often seen as the central focus of an interaction with a patient. Every assessment is followed by a risk form, and it seems to be written in nearly every subsequent clinical entry. In doing so, we have started to lose sight of what the patient themselves often thinks is important in the consultation, which is what really should be important to us. Yes, of course, we need to ask about and think about risk. But my concern is that a preoccupation with risk has distorted patient interactions and paradoxically may not make things safer.

**Nowadays, no kind of interview is complete without a question about artificial intelligence. What do you think artificial intelligence can bring to psychiatry?**

There is a utopian, or maybe a dystopian, future world in which artificial intelligence could reproduce exactly what a psychiatrist does. I have wondered whether intuition is nothing more and nothing less than just repeated experience. And if you showed an artificial intelligence around 100 000 or 100 million interviews, maybe it would have compassion, intuition and a great knowledge of psychiatry. I can’t see that happening, perhaps not in any future, but not in any near future. Artificial intelligence might be a useful adjunct within psychiatry, but I don’t see it taking on anything more than a peripheral role, or reproducing what a good psychiatrist would do, at least not on a timescale that is likely to concern me. And we often forget it is the human interaction, the sense of being really listened to and understood by another person, that can provide a real therapeutic benefit.

**I just want to finish by going back to your book. How was the experience of writing the book and, in the end, do you recommend that people do it?**

For me at least there are different phases. There is the having the idea, writing a proposal, then discussions with your agent and publishers, and that’s fun. Sitting and thinking about it and writing it in libraries, in cafes and everywhere, that’s fun as well.

And then there is a moment when the book goes out into the world, which I find terrifying, where you submit yourself to the scrutiny of your peers and the public. I find that for a few months it intrudes into my sleep. And then interviews like this and literary festivals I do enjoy.

**So you recommend it?**

Yes, but you need to know what you’re getting into.
